# The Effect of Humidity and UV Light Exposure on the Mechanical Properties of PA6 Matrix Reinforced with Short Carbon Fibers and Built by Additive Manufacturing

**DOI:** 10.3390/polym18020164

**Published:** 2026-01-07

**Authors:** Bernardo Reyes-Flores, Jorge Guillermo Díaz-Rodríguez, Efrain Uribe-Beas, Edgar R. López-Mena, Alejandro Guajardo-Cuéllar

**Affiliations:** 1 Escuela de Ingeniería y Ciencias, Tecnologico de Monterrey, Av. General Ramon Corona 2514, Guadalajara 45138, Jalisco, Mexicoedgarl@tec.mx (E.R.L.-M.); alejandro.guajardo@tec.mx (A.G.-C.); 2 Urrea Herramientas, Av. 5 de Febrero km 11, El Salto 45680, Jalisco, Mexico

**Keywords:** structural integrity, additive manufacturing, mechanical properties, polymer aging, UV, humidity

## Abstract

This work presents results of nylon-based composites used in additive manufacturing (AM) subjected to 24, 48, 96, 168, 336, and 504 h of continuous exposure to UV and 50% humidity. Sample coupons were built on a Markforged Two^®^ printer. To mimic UV exposure, samples were exposed to 253 nm UV light (UV–C), whereas for humidity, samples were placed at 50% relative humidity and 22 °C in a bi-distilled water atmosphere. The effects of said exposure were measured in tensile, Charpy impact energy, mass absorption, and Shore hardness D tests. Nylon gained 5.6% ± 0.48 mass after 504 h. For Charpy, absorbed energy went down from 0.463 J/mm^2^ to 0.28 J/mm^2^ at 504 h of humidity exposure. For Shore D, the variation goes from 59.1 ± 0.82 for zero exposure to 66.8 ± 2.5 at 504 h of UV exposure. Conversely, UV exposure induced an increase in Young’s modulus and Shore hardness, while significantly reducing impact energy to 0.32 J/mm^2^, indicating embrittlement confirmed by SEM analysis. FTIR analysis revealed hydrolytic degradation under humidity and photo-oxidative degradation under UV, affecting N–H and C=O bonds. These findings allow a designer to project the residual mechanical properties of a component up to its last day of service.

## 1. Introduction

One of the most popular techniques for polymers in additive manufacturing (AM) is material extrusion (MEX, material extrusion per ISO/ASTM 52900 [1], also known as fused deposition modeling, FDM) due to its affordability, speed, operational safety, and wide availability of materials such as ABS, PLA, PEEK, and nylon [2]. However, polymers used in MEX, such as nylon (PA6), are susceptible to degradation when exposed to adverse environmental conditions [3,4,5], which over time, negatively impacts their mechanical properties and limits the uses in engineering applications of these polymers [6,7] and polymer matrix composites (PMC) [8]. This paper explores the effect of UV and humidity exposure on PA6-based PMC mechanical properties. The results can provide a designer with a quantitative assessment of such variation over time and help forecast a component’s condition throughout its service lifetime.

Moreover, such degradation is widely documented in the literature [3,4,5,9,10,11], and consists of a change in mechanical properties such as hardness, a change in stiffness, and a decrease in ductility over time. The literature recognizes the problem of moisture absorption in nylon samples [7,12] whether submerged [8,13] or in a humid atmosphere [14]. Such mechanical tests have been reported for nylon reinforced with continuous fibers [8,15,16,17], 3D printed composites [3,4] and nylon matrix under other media [9]. Therefore, the degradation of AM printed polymers under different combinations of time, UV light, and humidity generates uncertainty over the service lifetime in the structural performance of components manufactured by this technique. However, damage in composites often starts at the matrix [18,19], but there are no studies for aging of just the MEX-printed PA6 matrix [13] due to the orthotropy imposed by the printing process [17,20]. It is important to know the effect of such exposure to understand such products’ long–term response [3].

Recent investigations focus on the hygrothermal aging and durability of composites with short carbon fibers [17]. Guo et al. [13] showed that the Onyx matrix reaches a saturation water content of 10.3% after 55 h immersed in water at 60 ± 1 °C. Then, they performed a three–point bending test where the general trend for flexural properties shows a gradual decline as aging effects become more pronounced. Shirinbayan et al. [14] performed tests to evaluate the physicochemical, thermomechanical, and mechanical properties of 3D-printed PA6, obtaining water saturation plateaus at different temperatures. However, hydrothermal conditions are not the only ones that pose a risk to the performance of polymers. UV light degradation in polymers has also been documented [12] as a failure mechanism for these polymers used as matrix. Moezzi et al. [21] conducted a study on the effects of UV degradation on the physical, thermal, and morphological properties of nylon 66 and [22,23] tested several polymers under UV exposure. They showed that UV exposure had a significant effect on the shear modulus of polyamide woven fabrics after as little as 20 h. They also found through FTIR and XPS that the shear modulus of the fabrics decreased after only 4 h of exposure, but it increased after 15 h. Again, the shear modulus was reduced by increasing the UV exposure time to 20 h [16]. Moazzami et al. documented flexural elastic modulus drop for CFRP and GFRP exposed to water at 50° [24]. Moezzi [21] focused on the breakdown mechanisms (photo-oxidation, chain scission, crosslinking, hydrolysis, and plasticization) caused by UV and humidity in PA6 and similar polyamides, including important chemical pathways and their effects on mechanical characteristics. However, in all these cases [3,5,13,21] a composite was tested. So, authors agree that damage in composites usually starts at the surface, hence the motivation for looking for changes in a matrix’s mechanical properties after exposure. This work explores the effects of UV–C and 50% humidity exposure at ambient temperature over time on a nylon matrix for AM composites. Table 1 shows a summary of testing conditions for relevant studies.

**Figure 1:**
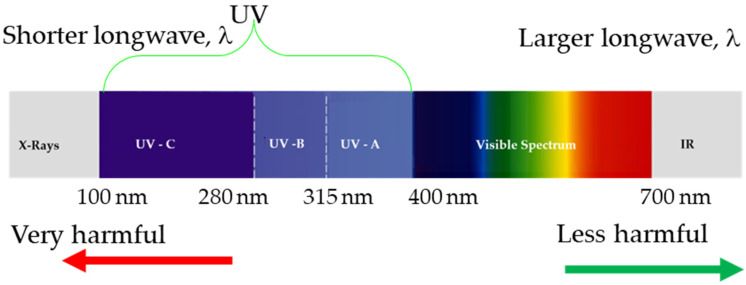
Electromagnetic spectrum.

What we call ultraviolet light (UV) is the wavelength portion of the electromagnetic spectrum between 100 and 400 nm approx., as depicted in Figure 1. The UV range is divided into three parts: UV–A (315–400 nm) is the least harmful for humans, while UV–B (290–315 nm) causes sunburn, increases the risk of skin cancer, and consequent cellular damage. About 95% of all UV–B light is absorbed by the ozone in Earth’s atmosphere, and UV–C (100–290 nm) is harmful but is almost completely absorbed by Earth’s atmosphere.

One way to optimize the mechanical properties of polymers is to embed short carbon microfibers into the thermoplastic matrix. The Mark Two^®^ printer uses carbon fiber reinforced nylon (Onyx^®^) with mechanical properties of an order of magnitude higher than common 3D printers [28]. However, it has been shown that variables in the FDM process, including the flow and thermal dynamics of the melt, the extrusion process, the bonding process between successive layers of material, temperature and printing pattern play an important role in how the final interface between printing beads is formed, which consequently affects the mechanical properties of the final part [18]. Components made from this material can be used as tooling/assembly/metrology fixtures and prototypes. This manuscript aims to have a better understanding of the effects on the mechanical properties of the Onyx material of relative humidity and exposure to UV light. The purpose of the study is to find out how mechanical properties are affected by exposure to 50% humidity and UV–C, both at ambient temperature but in separation. If the component is intended to operate under humidity or UV conditions, the designer must ensure it has mechanical properties which will last to the last day of service.

## 2. Materials and Experimental Methods

### 2.1. Material

Nylon is a synthetic polymer composed of repeating amide groups [29], forming strong covalent bonds that give it flexibility and toughness. Nylon resists various oils and greases but can degrade with strong acids and bases [9]. The repeating amide groups attract water molecules, which causes a tendency of the material to absorb water leading to dimensional swelling and softening [6,7]. Nylon has a melting temperature of 250 °C and has been used for AM due to its strength, toughness, versatility, and ease of processing by different AM techniques.

To improve nylon’s performance and finishing, short carbon fiber can be added to the matrix resulting in a composite. Carbon fiber is highly resistant to most chemicals, including acids, bases, and organic solvents, and can withstand high temperatures. In the resulting composite, the components are not bonded chemically; the fibers are dispersed in and held in place by the matrix.

This polymer is used as matrix for 3D printed composites used for tooling, grips for low batch production and assembly/metrology fixtures. Between usage and due to storage, such applications may be subjected to UV and humidity exposure, hence the motivation for this study. Other uses reported are low–cost but customized prostheses, and prototypes.

### 2.2. Sample Preparation

**Figure 2:**
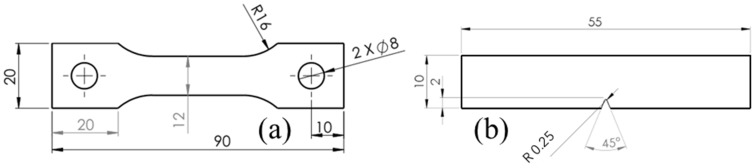
Testing samples geometries: (**a**) tensile, (**b**) impact. Dimensions in mm.

Samples were fabricated using the MEX technology (per ASMT/ISO 52900 [1]) using a Mark Two printer (Markforged^®^ Inc., Waltham, MA, USA), with Onyx filament, which was also supplied by Markforged^®^. It has a density of 1.2 g/cm^3^, tensile modulus of elasticity of 2.4 GPa, and a tensile stress of 40 MPa [30]. A total of four samples per treatment were printed with a solid fill pattern and were designed according to ASTM D638 [31] for tensile tests, 4 mm thick, as shown in Figure 2a, and ASTM D6110 [32] for Charpy, 8 mm thick, as shown in Figure 2b. Kikuchi [17] concluded that this Nylon is PA 6.

#### 2.2.1. Test Conditions

The tests included two environmental conditions: Relative humidity and exposure to UV–C light, each conducted with the two types of specimens shown in Figure 2, impact and tensile. Due to latitude and environmental conditions, Mexico has a high UV index [33]. Therefore, it was decided to perform an accelerated test by exposing samples to shorter wavelength but with higher energy as suggested in the recent literature for other polymers [22,26,27]. Exposing the samples to higher power can speed up photodegradation to assess long–term behavior in shorter test times [34]. It also helps with rapid stability testing to understand potential failure modes. Furthermore, Bergeret et al. [8] discussed how accelerated aging, under harsher conditions, helps understanding of polymer behavior without significantly delaying product development. Using conservative values for the efficiency of the lamp, the daily UV radiation dose exposure of the sample estimated is 71,158.09 Wh/m^2^. For each test condition, four specimens of each type were used in each of the exposure periods: 24, 48, 96, 168, 336, and 504 h; plus, the control group that was not exposed to any conditions after printing. ASTM D618 [35] recommends 50% humidity exposure at 23 °C for at least 96 h. This scheme allowed for consistent comparison across both testing environments and exposure times, ensuring robust data on the material’s performance while looking at long term degradation. Furthermore, sample coupons were tested for tensile, impact, hardness, SEM, and FTIR. Such testing provides an assessment of UV and humidity-induced degradation: structural, mechanical, and chemical modifications, providing a comprehensive understanding of the material’s deterioration and its response to adverse environmental conditions over time. Details of each experiment are as follows.

#### 2.2.2. Humidity Conditions and Water Absorption Methodology

The humidity conditions were created using an ACE UV Accelerated Weathering Test Chamber, model UV–260 (LIB, Xi’an, Shanxi, China), at conditions of 50% relative humidity at 22 °C using bi–distilled water to avoid possible contamination by foreign ions.

To measure the water absorption of the material, first, the initial mass of the samples was measured using an analytical balance (Pioneer PX OHAUS, Darmstadt, Germany) with readability of 0.001. Samples were extracted every 24, 48, 96, 168, 336, and 504 h. The percentage moisture content Mt is defined as shown in Equation (1):

(1) $$M_{t}=\frac{W_{t}-W_{i}}{W_{t}}$$

where *Wt* is the weight of the wet material at moment *t*, and *Wi* is the weight of the dry material.

#### 2.2.3. UV

A closed environment was designed and custom–built for UV light exposure using 253.7 nm wavelength UV, type C, 25 W commercial lamps. The specimens were subjected to the same exposure time and continuous radiation to ensure comparability between both conditions. Therefore, in this manuscript, henceforth UV refers to UV–C. However, the lamp intensity was not monitored over time. Applications for that wavelength include, but are not limited to UV curing, metrology and material processing, laser medical treatment, and scientific experiments.

### 2.3. Impact and Hardness Testing

Hardness was measured with a TIME Group Inc. durometer, model TH210 (Maastricht, The Netherlands), using a Shore D scale, according to the ASTM D2240 [36] standard, with an applied load of 49 N. The minimum sample thickness of 6 mm per ASTM D2240 was met.

To assess specimen impact toughness after various exposure durations, an ITC–300 impact pendulum from Physical Test Solutions (Jinan, Shandong, China), calibrated with a 150J hammer, was used. Testing was conducted in compliance with the ASTM D6110 [32] standard.

### 2.4. Tensile Testing

Tensile strength and elongation tests were performed using a Galdabini QUASAR 50 universal testing machine (Cardano al Campo, Lombardia, Italy), with a 50 kN load cell capacity, at a 2 mm/min rate following ASTM D638 [31]. Control and data acquisition was done with Labtest 3.1 software at a 10 Hz rate. From that, the stress vs. strain curve was built, and Young’s modulus, and maximum strength were extracted.

### 2.5. FTIR

**Figure 3:**
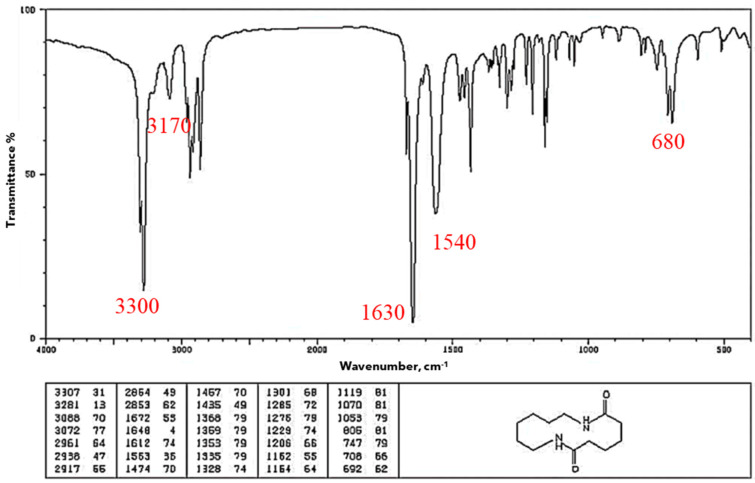
FTIR pattern for nylon [37].

Fourier transform infrared spectroscopy (FTIR) was employed to analyze the material samples by identifying functional groups and chemical bonds present in the material. FTIR is a helpful technique for figuring out how a polymer’s degree of crystallinity has changed after a particular treatment [22]. The analysis was conducted using a Shimadzu IRAffinity–1S spectrometer(Kyoto, Japan) in ATR mode (Attenuated Total Reflectance) equipped with a diamond crystal. Data acquisition was done with LabSolutions IR software V 2.1 from Shimadzu. The instrument performed 40 scans for each sample, to average results and ensure accuracy. The characteristic spectrum for nylon, in absorption mode, is shown in Figure 3 [37] where one can see its characteristic bands: O–H 3300, N–H 3170–3370; 1515–1570; 680–750, C=O 1630–1680 cm^−1^. This is the baseline that later was used to evaluate Onyx as-is and Onyx after the humidity and UV treatments. It is important to clarify here that we did not find the FTIR pattern for Onyx, but there are several FTIR patterns for nylon [38].

### 2.6. Microscopy

A JEOL IT 700 HR (Tokyo, Japan) scanning electron microscope (SEM) was utilized to perform fracture surface and microstructural analysis of the samples. The microscope was operated at 3 kV. The short carbon fiber reinforcement embedded within the matrix made the sample conductive, so no gold plating was required. Analysis provided surface morphology, fracture features, and any microstructural changes that may have occurred due to the experimental variables. This technique is useful for identifying failure mechanisms, such as fiber pull–out, matrix cracking, or interfacial debonding, which are critical for understanding the material’s performance and degradation.

## 3. Results

### 3.1. Moisture Absorption

**Figure 4:**
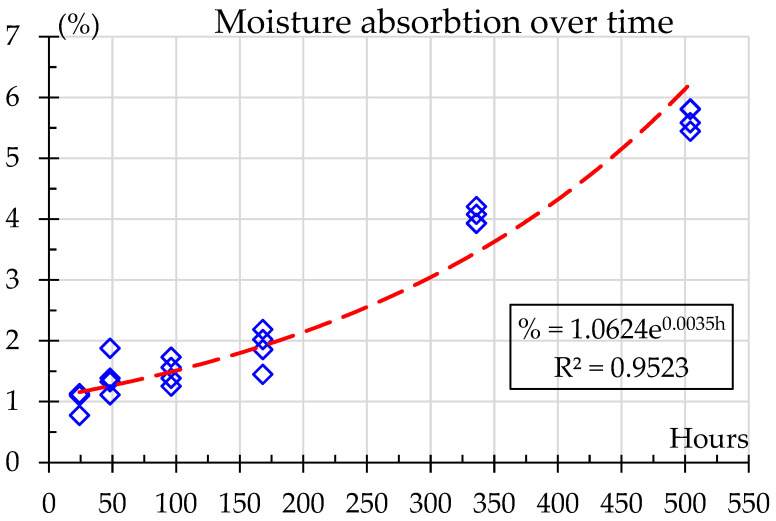
Moisture absorption over time.

The absorbed humidity content in the material over the exposure times, from 0 to 504 h, is shown in Figure 4. A clear rising trend is observed, indicating a gradual increase in moisture absorption as exposure time progresses. At 24 h, the moisture content is relatively low, around 1%, increasing steadily over the first week, reaching up to 2%. The most significant rise occurs after 168 h, eventually climbing to around 5.6% after 504 h. This steady increase suggests that prolonged exposure leads to higher moisture absorption and agrees with Bergueret et al. [8] who showed water saturation, around 9% in weight, after as little as 30 h; however, their samples were immersed in water at 135 °C. The results show that saturation is not reached; however, for exposure times greater than 168 h the percentage moisture absorption decreases over time as expected in a diffusive-like behavior prior to reaching saturation. For this study, moisture absorption over time can be accurately predicted using the exponential relation shown in Equation (2).

(2) $$\%=1.0624e^{0.0035h}$$

where % is the mass gain percentage, and h is the exposure time in hours. This model provides a high degree of predictability, as evidenced by an R^2^ = 0.9523, indicating that over 95% of the variability in moisture absorption can be represented by this equation. The adjusted R^2^ is very close, 0.9477, showing that there are no redundant data. The adjusted R^2^ penalizes the addition of irrelevant independent variables, providing a more accurate measure of the model’s explanatory power than R^2^ [39].

### 3.2. Impact Results

**Figure 5:**
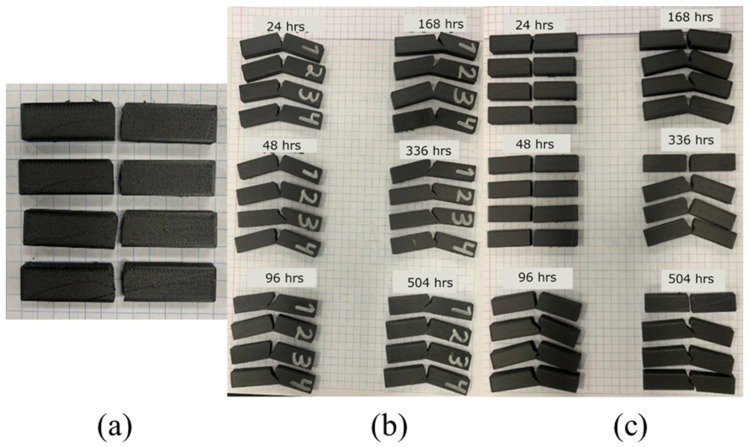
Morphology of samples after impact testing; (**a**) control, (**b**) 50%, humidity (**c**) UV.

The control samples, for impact testing, are shown in Figure 5a. One can see that all of the samples broke with a clearly defined and straight path. On the other hand, none of the humidity-exposed samples broke completely, with a residual ligament keeping the sample together, as shown in Figure 5b. This partial breaking behavior has been documented before for room [18] and other temperatures [40], which may indicate that humidity did not change impact behavior. On the other hand, UV samples showed partial breaking behavior after 96 h of exposure. Furthermore, for the most part, the crack path followed the direction perpendicular to the maximum normal stress as induced by the applied load. In cases where it did not (mostly in the humidity-exposed samples), deviation from the preferred path might be attributed to the intrinsic anisotropy induced by the printing process. Garcia et al. [18] also noted that cracks started perpendicular to the notch but in some instances deviated from that direction, and Guo et al. [25] reported how humidity also affects fatigue performance and crack propagation, especially mode II which dictates how a crack kinks.

**Figure 6:**
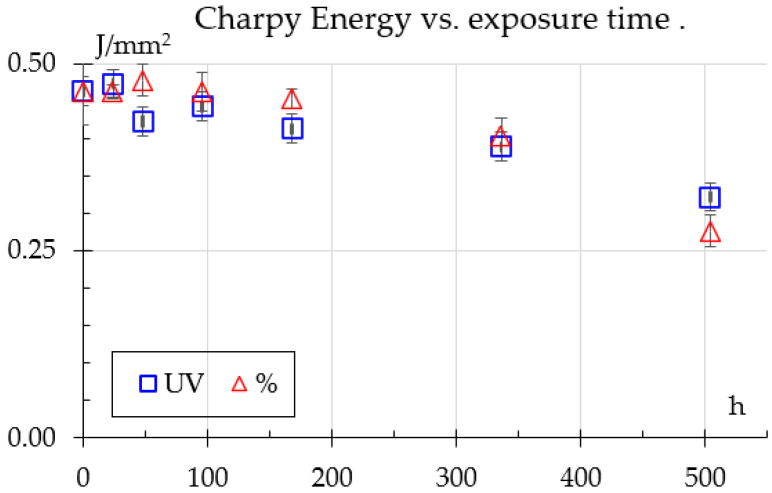
Charpy impact results.

Charpy tests are shown in Figure 6. For humidity samples, energy absorption rises and remains about the same up to 96 h, followed by a gradual decline, indicating moisture-induced weakening in the material. In contrast, UV exposure causes an initial increase in energy absorption, peaking at 24 h, but rapidly declining after 96 h, reaching very low levels at 504 h. This highlights that both UV and humidity exposure induce severe material degradation, likely due to surface effects or cross–linking.

The impact results show a similar trend to what Bergueret et al. [8] reported, even showing a peak after initial exposure to humidity falling to about 12% below the unexposed samples; however, they tested only until 270 h.

### 3.3. Hardness Testing Results

**Figure 7:**
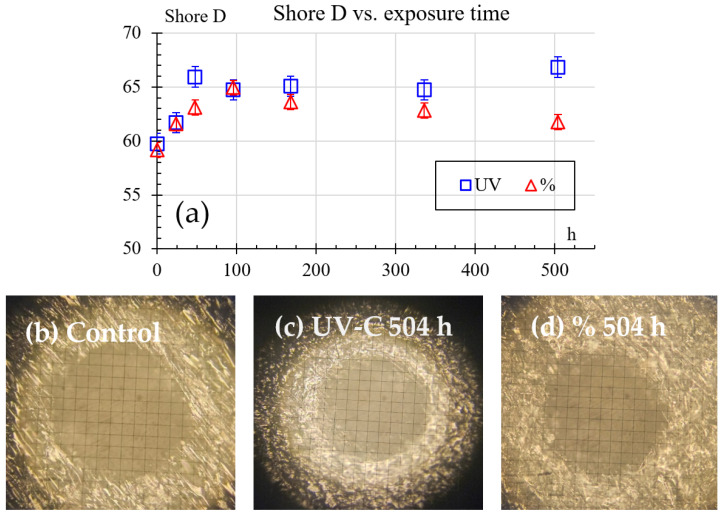
Shore D hardness results: (**a**) hardness vs. exposure time, (**b**) exemplary result of imprint with no exposure, (**c**) exemplary result of imprint after 504 h of UV exposure, (**d**) exemplary result of imprint after 504 h of 50% humidity exposure.

Hardness Shore D results are shown in Figure 7, exhibiting an initial average value of 59.7. This value rises in the first 96 h, reaching a peak of 65.9 and 64.9 for the humidity and UV samples, respectively. After 96 h, the hardness tends to decrease. Humidity samples at 504 h of exposure reach a value of 61.8. However, UV samples at 504 h of exposure reach a maximum value of 66.8. For UV conditions, this behavior indicates an inverse relationship between hardness and impact resistance. Increased Shore D hardness corresponds to decreased Charpy energy absorption, suggesting that UV exposure makes the material harder but more brittle. In contrast, humidity exposure has a less pronounced effect on hardness, but it gradually reduces impact resistance over time, likely due to moisture-induced weakening of the material’s internal structure. Furthermore, there are exemplary results of indentation size with no exposure, as shown in Figure 7b, after 504 h of UV exposure in Figure 7c, and after 504 h of humidity exposure in Figure 7d. Finally, the exemplary indentation photographs show that the indentation imprints were done in a defect-free area and their size is not near the size of the material’s structure, validating the measurements.

### 3.4. Tensile Testing

**Figure 8:**
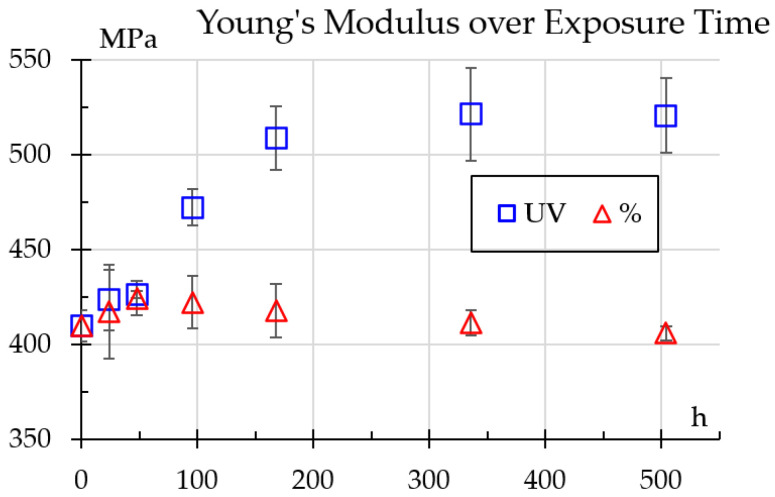
Young’s modulus values change over exposure time.

Figure 8 illustrates progression of Young’s modulus values for samples exposed to UV light and humidity. One can see distinct trends for each treatment. For UV-exposed samples, the modulus steadily increases during the first week and remains stable. Its evolution was adjusted to the time-dependent relation shown in Equation (3).

(3) $$E=298.28h^{0.0908}$$

This increase suggests surface hardening and cross-linking effects, increasing stiffness but potentially leading to brittleness, as shown previously in the impact and hardness testing results [3]. On the other hand, samples exposed to humidity exhibit a slight decline, adjusted to the relation shown in Equation (4). As a comparison, Shirinbayan et al. [14] reported an elastic modulus decrease of about 25%, but for samples submerged in water.

(4) $$E=479.22h^{-0.027}$$

These findings demonstrate how UV and humidity trigger opposing degradation mechanisms, significantly influencing the long–term mechanical performance of the material. Furthermore, these results agree with the authors of [4] who documented the influence of humidity on flexural rigidity change. In comparison, Shirinbayan et al. [14] reported a 20% drop in strength for samples fully submerged in water whereas [8] reported that tensile strength fell to half after as little as 30 h, but samples were immersed in water at 135 °C.

**Figure 9:**
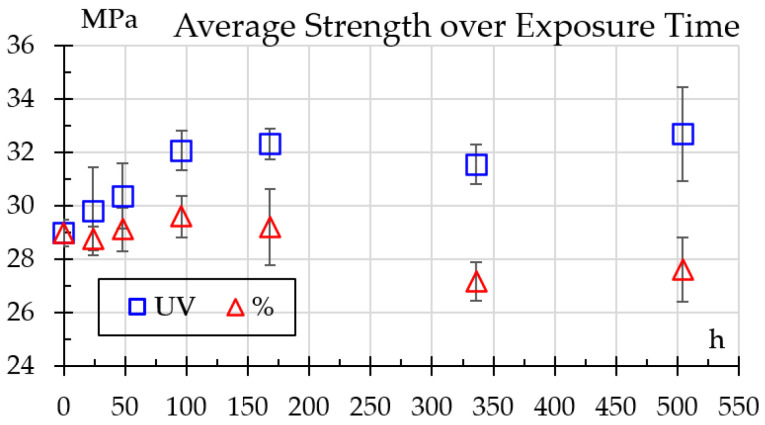
Strength variation over exposure time.

Results for tensile testing are shown in Figure 9. One can see that strength steadily rises with UV exposure from 29 ± 0.51 MPa to 33.6 ± 0.53 MPa at 504 h, whereas for humidity exposure it presents a little increase—as high as 30.6 at 96 h—but drops to 27.4 MPa at 504 h.

Tensile results tend to agree with elastic modulus results as both tests measure mechanical properties reflecting how the polymer responds to external loading, albeit measuring a different response. A longer polymer tends to be more rigid, which affects its resistance to plastic deformation. Because it takes more effort to deform or break the chains, this increase in rigidity may lead to a greater resistance to tension.

However, the relationship is not always linear: some strong polymers may be brittle and easily break (having little tenacity or tensile strength), while other, less strong ones may be more resilient and resistant to testing due to their ability to absorb energy before breaking. Both properties are simultaneously impacted by the molecular stiffness, length, and encrustation of the polymer chains and intermolecular forces. For instance, increasing molecular weight and the number of intermolecular bonds often improves strength and usually hardness.

### 3.5. FTIR

**Figure 10:**
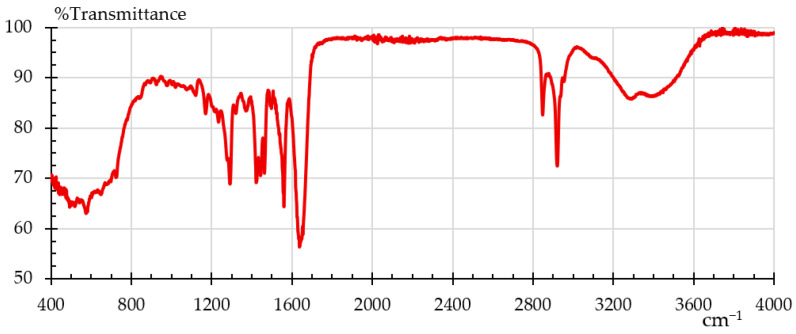
FTIR pattern for Onyx.

Figure 10 depicts the FTIR spectra obtained from the Onyx sample as it is, whereas Figure 3 shows results for PA6 [37]; one can see that both exhibit very similar patterns. It is assumed that because the short carbon fibers are so small (8 µm in diameter and 100 µm in length [41]) and in random orientations, they do not interact with the incident IR wave, becoming transparent to it. Therefore, the effect of the chopped carbon fiber embedded in the matrix does not significantly alter the FTIR spectrum.

**Figure 11:**
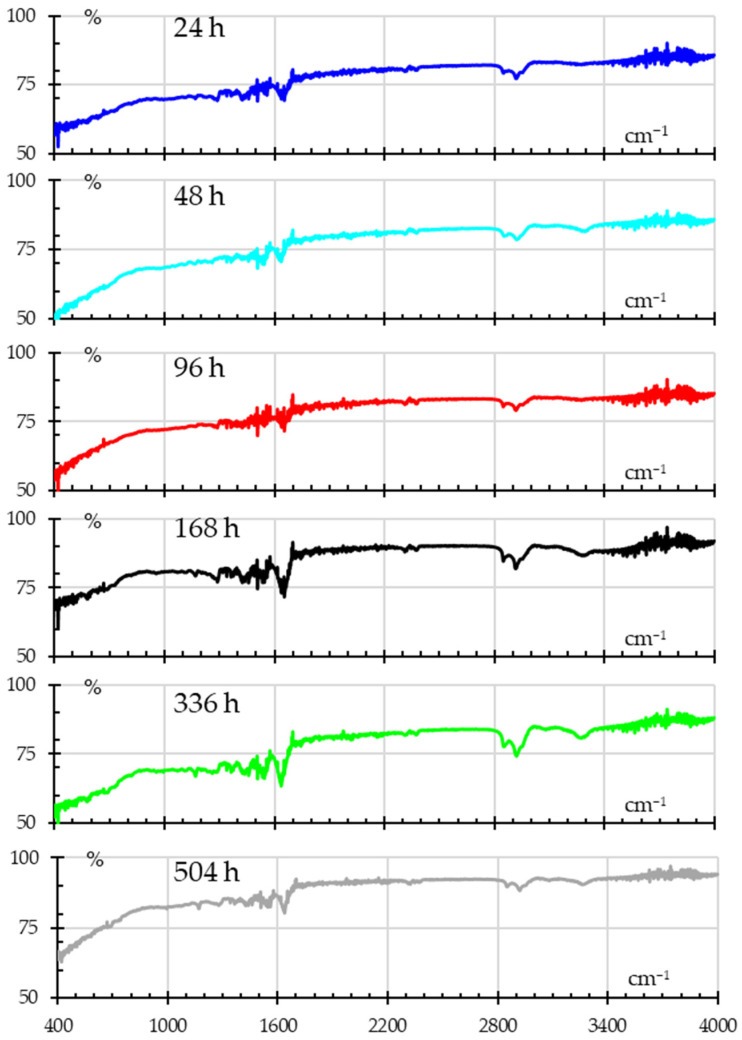
FITR spectra for samples subjected to different humidity times.

Figure 11 shows the absorbance spectra for samples subjected to humidity, showing how the continuous and extended exposure to humidity alters the chemical structure of nylon. The peak observed around 3000–3300 cm^−1^ corresponds to the N–H stretching vibration, whilst the O–H stretching region shows from 3200–3600 cm^−1^. Peaks from 2800 cm^−1^ to 2950 cm^−1^ are attributed to the stretching of –CH_2_– groups. The broad peak at 1640 cm^−1^ is due to C=O stretching (Amide I), while the peaks around 1550 cm^−1^ are associated with N–H bending and C–N stretching (Amide II) [37,38,42]. Finally, one can see the band between 1800 to 2800 cm^−1^ is transparent to the incident IR wave.

In the first 96 h, minimal changes are observed in the spectra, with only slight broadening in the O–H stretching band (3200–3600 cm^−1^). This suggests initial moisture absorption but no significant chemical alterations in the polymer structure. After 168 h, the increased O–H peak intensity indicates a high level of moisture absorption, likely penetrating deeper into the material as exposure time continues, which coincides with the mass absorption tests. At 336 and 504 h, small changes in the amide and carbonyl region (N–H and C=O) hint at hydrolysis and hydrolytic degradation, breaking down amide bonds in the polymer. This degradation can lead to the formation of new carbonyl-containing groups, which weaken the material. Also, in the fingerprint region (500–1500 cm^−1^), there is noticeable broadening after 168 h, which may hint at changes in the polymer’s microstructure.

The following is a summary of the spectral evolution.

1. There are minor variations in the absorbance values throughout the spectra over the first 24 to 96 h of humidity exposure. These could point to subtle interactions between the functional groups of nylon and water molecules. The existence of O–H stretching from absorbed water is often indicated by increases in absorbance around 3300–3500 cm^−1^;
2. The FTIR spectrum exhibits an increase in peak intensities after 168 h, indicating increased water absorption and possible nylon material expansion. This may be due to how water interacts with the amide groups (about 1640 cm^−1^ and 1500 cm^−1^), signifying H bonding and physical alterations in the nylon;
3. Considerable water absorption is indicated by notable variations in the spectra for the 336 and 504 h exposures. Peaks that correspond to amide I (C=O stretching) at around 1640 cm^−1^ and O–H stretching at about 3300–3500 cm^−1^ intensified. This would suggest that the water molecules and the nylon polymer chains have formed a stronger hydrogen-bonded network. Furthermore, modifications in the 600–800 cm^−1^ area (linked to the C–H bending vibrations) may indicate changes in the molecular interactions within the polymer matrix because of extended exposure to water.

All things considered, the spectra demonstrate how prolonged exposure to humidity progressively changes nylon’s chemical structure, mostly because of interactions between water molecules and the functional groups of the polyamide. This affects mechanical properties like strength and elasticity.

**Figure 12:**
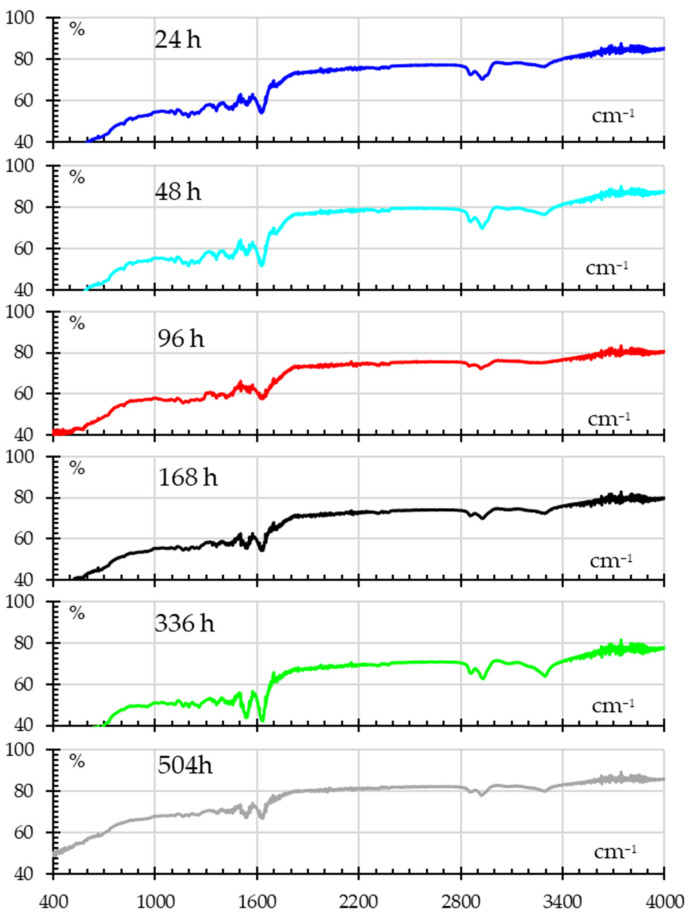
FITR spectra for samples subjected to different UV exposure times.

The FTIR spectra for UV-exposed samples are shown in Figure 12, showing signs of photodegradation. Shifts in the N–H bending and C–N stretching regions (around 1500–1600 cm^−1^) after the first 24 h indicate possible alterations to amide bonds that show progressive change. The O–H and C=O regions continue to show increased absorbance at 336 h, suggesting that prolonged UV exposure causes oxidation and the formation of new hydroxyl and carbonyl groups.

A summary of changes is as follows.

1. The spectra show slight variations in absorbance throughout the first 24 to 96 h of UV exposure. Early stages of photodegradation, in which UV radiation breaks chemical bonds in the nylon and modifies peak intensities slightly, may be the cause of these changes. The production of carbonyl groups (C=O stretching), which are common by-products of photodegradation, may be indicated by modest increases or shifts in absorbance around 1700 cm^−1^;
2. There are noticeable alterations in the spectra at 336 and 504 h, indicating significant photodegradation. Significant increases in the carbonyl peaks’ intensity (~1700 cm^−1^) indicate a substantial buildup of degraded byproducts, agreeing with Rajendran [23]. Changes in the O–H stretching areas about 3300–3500 cm^−1^ could be a sign that hydroxyl-containing groups are forming. Changes may also be seen in areas linked to C–H stretching (~2900 cm^−1^) and bending (~1400 cm^−1^), which demonstrate the disintegration of the polymer backbone and the creation of smaller molecules;
3. There are noticeable alterations in the spectra at 336 and 504 h, indicating significant photodegradation. Significant increases in the carbonyl peaks’ intensity (~1700 cm^−1^) indicate a substantial buildup of degraded byproducts. Changes in the O–H stretching areas about 3300–3500 cm^−1^ could be a sign that hydroxyl-containing groups are forming. Changes may also be seen in areas linked to C–H stretching (~2900 cm^−1^) and bending (~1400 cm^−1^), which demonstrate the disintegration of the polymer backbone and the creation of smaller molecules.

In general, nylon’s chemical structure deteriorates with extended UV exposure, mostly due to the creation of carbonyl and hydroxyl groups. Nylon’s mechanical qualities are impacted by this deterioration, which over time makes it less resilient and more brittle. The findings emphasize the substantial degradation of nylon caused by UV radiation, as well as the alteration in mechanical characteristics following extended exposure to UV light.

### 3.6. Electron Microscopy

**Figure 13:**
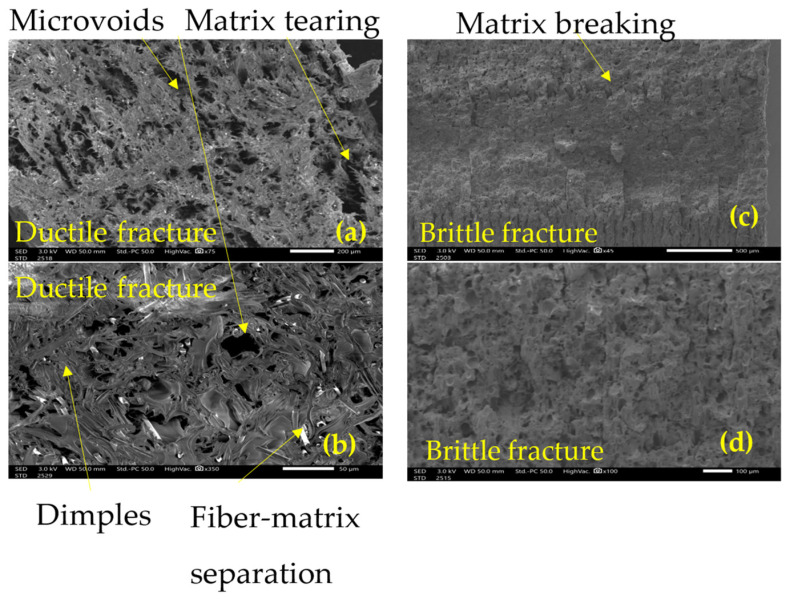
Failure surface at 0 hr exposure: (**a**) tensile x75, (**b**) tensile x350, (**c**) impact x45, (**d**) impact 100x.

The SEM analysis of tensile and impact tests provides clear evidence of the degradation mechanisms induced by UV exposure by showing the fracture surface. At 0 h, the tensile tests reveal a fractured surface typical of ductile failure mode as seen in Figure 13a, characterized by visible deformation and high energy absorption by means of matrix–fiber debonding and matrix rupture [43] as seen in the rough, fibrous texture depicted in Figure 13b. Such behavior has been reported for the same matrix [17], from a different provider and for different printing layer height [44]. The matrix exhibits a cohesive interaction with the filaments, allowing significant plastic deformation before rupture, characteristic of a ductile material able to withstand high energy absorption [43]. These are signs of plastic deformation and possible indications of chains stretching in the polymer [43]. The impact testing induces a high strain rate; therefore, the failure mode is dominated by rapid crack propagation and a lack of significant deformation in the filaments, as shown in Figure 13c,d, where the characteristics of brittle fracture can be seen. The load is applied very rapidly, so the material does not have time to accommodate and absorb energy, producing an unstable rupture [45].

**Figure 14:**
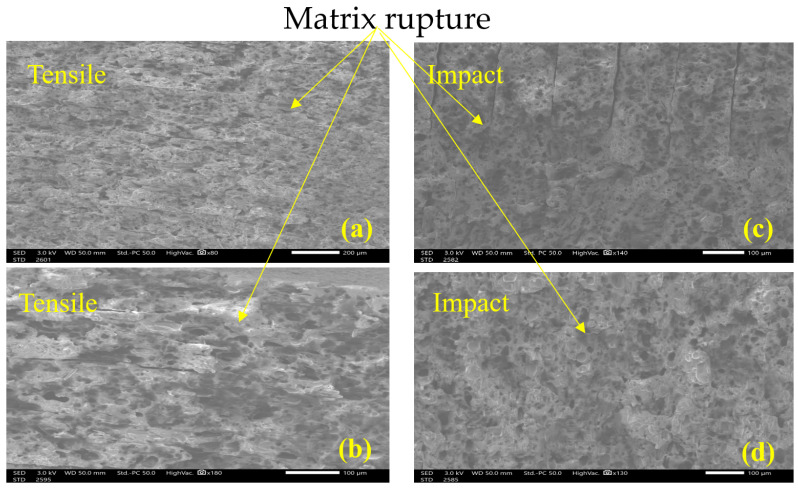
Failure surface at 504 h UV exposure: (**a**) tensile x80, (**b**) tensile x180, (**c**) impact x140, (**d**) impact 130x.

In contrast, after 504 h of UV exposure, the tensile samples exhibit a drastically different fracture behavior. The surface shows signs of embrittlement in the matrix as seen in Figure 14a, which limits the ability of the Onyx filaments to absorb energy while the quasi-static load is applied. Instead of the characteristic stretching and tearing observed in ductile failure, the matrix cracks and ruptures cleanly as seen in Figure 14b, transferring stress more abruptly to the filaments resulting in a reduced ability to absorb energy. This brittle failure mode is marked by smoother and more planar fracture surfaces when compared to samples with no UV exposure.

## 4. Discussion

When R^2^ and adjusted R^2^ are close, it could mean that the model is not too complicated and that the model’s fit to the data would not be improved by the addition of more independent variables beyond what would be predicted by chance, as shown in Figure 4. However, there is a saturation point [17] where the material can no longer absorb moisture. Further tests are needed to determine the saturation point of the material under aging conditions [4].

Guo et al. [13] showed that the Onyx matrix reaches a saturation water content of 10.3% at 55 h under temperature-controlled water bath at 60 ± 1 °C [6]. Then, they performed a three-point bending test, where the general trend for flexural properties shows a gradual decline as aging effects become more pronounced. This condition causes water to get into the middle of the fiber matrix, reducing the adhesion of fiber to matrix, making any fiber more susceptible to interface debonding or pulling out. Bergeret et al. [8], on the other hand, showed saturation after as little as 30 h but for samples fully immersed in water at 135 °C. Shirinbayan et al. [14] showed saturation after as little as 75 h approx., but for submerged samples at 70 °C. In the tests performed, saturation was not observed similar to what Humeu et al. [7] reported at room temperature. Bergueret et al. [8] discussed two possible mechanisms: that water forms a double hydrogen bond with the carbonyl groups (1640 and 1850 cm^−1^) or it is loosely bound with other polyamide bonds; but such discussion is out of scope of this paper. Finally, samples were not dried after FTIR testing to verify that this study could be performed in the future. So, we see a direct relationship between water absorption and temperature for hygrothermal degradation.

Elasticity (Figure 8) and strength (Figure 9) showed similar trends. This could be attributed to photochemically induced reactions that generate crosslinking in the polymer, increasing strength and elasticity, especially in the initial stages or under controlled conditions. Amide bonds (between the carbonyl group (C=O) and the amino group (NH_2_)) in nylon can absorb UV energy, which can cause them to break and lead to the formation of free radicals. Moezzi [21] reported loss of elasticity after as little as 4 h exposure but a gain after 20 h, attributed to cross-linking or polymer fusion that fills cracks thus improves stress resistance. In some cases, this is also associated with a change in color [23]. Although color change was not measured, such behavior has been reported for nylon [34]. This could be attributed to short carbon fibers that were not sufficiently exposed to generate such discoloration.

Except for the Shore D hardness test, all mechanical tests gave lower values for humidity exposure. This phenomenon is to some extent reversible. Drying nylon samples, at least to some extent, restores mechanical properties. It is a common practice to dry out not only nylon but other polymers commonly used in FDM as well, when left in humid environments [17]. This process improves printability.

On the other hand, SEM showed characteristics of brittle failure after exposure, corroborating the decrease in tensile strength and impact on energy. Samples exposed to UV after a static test showed the same brittle morphology as impact samples (see Figure 12). It has been suggested [21] that UV light causes photo-oxidation in nylon, weakening the polymer structure and causing cracks and internal damage visible under microscopy. However, the SEM analysis was not able to identify such cracks. Such embrittlement has been reported for O_2_ diffusion [46]. This damage compromises the nylon’s ability to absorb energy during impact, making samples appear brittle much like mechanically impacted samples that have undergone fracture [21].

**Figure 15:**
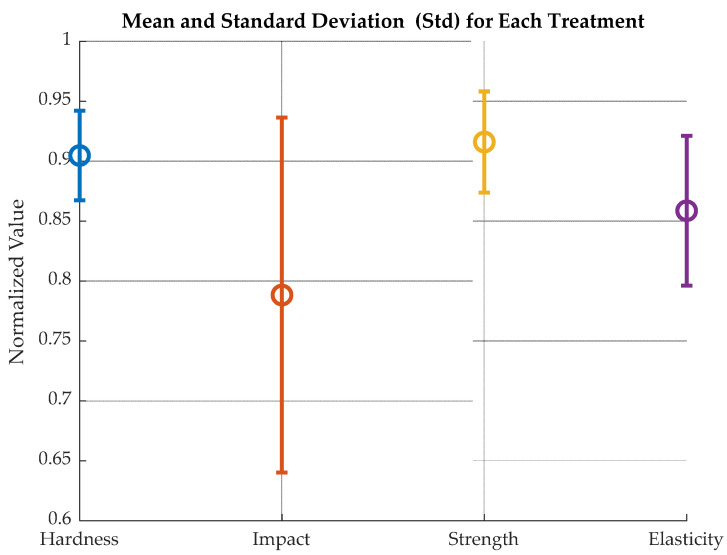
Normalized mean and standard deviation for humidity-exposed samples.

A one-way ANOVA was run with normalized values to assess the difference between treatments with an uncertainty of 5%. Normalized values refer to Strength, Elasticity, Hardness, and Impact values divided over their respective maxima. The null hypothesis was that “the averages of the different measurements are equal among them”. Figure 15 shows the comparison of normalized values for mean and standard deviation and Table 2 the relationship among variables.

The results of comparing mean and standard deviation among treatments are as follows:

Impact Charpy vs. Hardness Shore: *p* = 0.00000 (significant)

Impact Charpy vs. Strength: *p* = 0.81626 (not significant)

Impact Charpy vs. Young: *p* = 0.00185 (significant)

Hardness Shore vs. Strength: *p* = 0.00000 (significant)

Hardness Shore vs. Elasticity: *p* = 0.00017 (significant)

Strength vs. Elasticity: *p* = 0.00356 (significant)

This means that normalized average values for strength, Elasticity and Shore D hardness are statistically equal. This suggests that the observed differences between normalized values may be due to manufacturing differences, treatment variability or testing error within groups, and not to a real effect of the factors analyzed.

**Figure 16:**
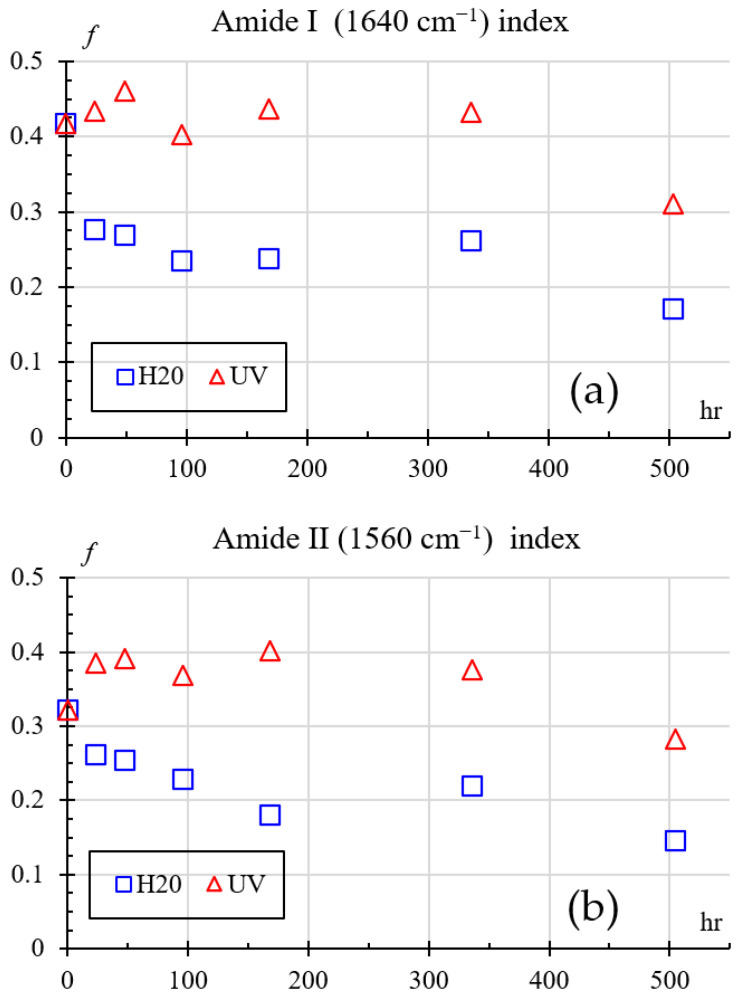
Amides index change: (**a**) Amide I (1640 cm^−1^), (**b**) Amide II (1560 cm^−1^).

Amide groups are monitored mainly through amide I (around 1640 cm^−1^) and amide II (around 1560 cm^−1^) bands. Changes in these peaks reveal details of the polymer chain backbone structure such as chain scission [34], crystallinity changes, or hydrogen bonding alterations between N–H and C=O groups. Therefore, to quantify nylon’s degradation through FTIR, the index shown in Equation (5) is used.

(5) $$f=\frac{I-I_{ref}}{I_{ref}}$$

where *I* is the intensity at a given wavelength, *I_ref_* is the intensity at 2400 cm^−1^, and *f* is the fraction change. The 2400 cm^−1^ wavelength was chosen because it is transparent to IR and was unaffected in all treatments. Figure 16 shows how the amines change over time for both exposures, Figure 16a for 1640 cm^−1^, and Figure 16a for 1560 cm^−1^. Comparing the indexes with respect to the baseline (0 h at 41.78%), one can see that both amide indices show variations from 3 to 6% for UV and 23% for humidity exposure during the first 24–96 h. This behavior is consistent with an early stage of chemical change in which only limited modification of the amide environment occurs.

At intermediate times (around 168–336 h), the Amide I and Amide II indices for UV-exposed samples remain relatively high. For water-exposed samples the index decreases, indicating that UV tends to preserve or slightly reinforce the amide bands through surface crosslinking, whereas humidity promotes progressive disruption of hydrogen bonding and partial hydrolysis of amide groups.

Finally, after 504 h, both indices drop for the UV-exposed samples and continue to fall for the water-exposed samples, showing that prolonged aging (UV or humidity) ultimately reduces the amide content or its effective contribution to the spectra, which is consistent with chain scission and the formation of new oxidized or hydrolyzed species.

The intermediate peak in the mechanical properties shown in Figure 6, Figure 7 and Figure 9, followed by a decrease, is not a phenomenon unique to this study and is attributed to competing internal processes. Moezzi et al. [21] found that for PA66 the shear modulus did not show a uniform trend. This phenomenon was attributed to the fact that exposure to UV light causes the breakdown of polymer chains (fragmentation/cleavage and scission of the chain) and the formation of free radicals. However, there is competition between chain scissions and crosslinking. Chain scission in polymers causes a significant drop in their mechanical properties, while their crosslinking causes a significant increase in these properties. The temporary increase in the mechanical properties of the UV-exposed samples suggests hardening of the surface and cross-linking effects. This hardening correlates with a decrease in the impact strength (embrittlement).

In the same Figure 6, Figure 7 and Figure 9, but under the effect of 50% RH, the non-uniform variation in mechanical properties can be influenced by plasticization (water content reduces the Glass Transition Temperature, which softens the material) and, therefore, its microstructural reorganization. Guo et al. found similar results and discussed how exposure to moisture can promote crystal growth and molecular reorganization. This process can temporarily increase the strength and flexural modulus (although the modulus of elasticity in tension was measured in this study, the two measurements are comparable). However, continued exposure of the polymer to moisture caused subsequent hydrolysis, causing these properties to decrease again.

The observed shifts and changes in the FTIR peak positions, particularly in the hydroxyl and amide regions, align with findings from [14,21]. Comparable research has demonstrated that variations in hydrogen bonding, as reflected by shifts in the O–H stretching region, serve as reliable indicators of changes in the local environment of hydroxyl groups. Likewise, alterations in the amide I and amide II bands, around 1650 cm^−1^ and 1540 cm^−1^, respectively, have been consistently reported [21] to correlate with modifications in polymer backbone integrity, including chain scission and changes in crystallinity. These studies similarly emphasize that monitoring these bands provides insight into hydrogen bonding dynamics between N–H and C=O groups, which affect the overall polymer structure. The consistency of spectral changes reinforces the utility of FTIR as a tool for detecting molecular interactions and structural rearrangements in polymers giving reliable evidence that links alterations in hydrogen bonding and crystallinity which govern material properties [8]. Furthermore, [10,11] deeply discussed photodegradation of polyamide which primarily occurs through ultraviolet (UV) radiation, which excites the polymer molecules, leading to bond cleavage and chain scission. The process involves several pathways, including the dissociation of hydrogen radicals from nitrogen or carbon atoms, and the cleavage of key bonds within the polymer chain, such as the C−N bond in the peptide group and the N−C bond adjacent to the carbonyl group. These bond disruptions can occur via excited-state transitions in which electrons shift from lone–pair orbitals to antibonding orbitals, facilitated by conical intersections (CIs) between the ground and excited states. Internal conversion through these CIs leads to bond breakage and the formation of radical species, which propagate degradation reactions [10,11].

In other instances, polymer has become brittle by exposing samples to low temperature [40]. In this case, the exposure to UV–C light produced a decrease in impact strength. Furthermore, in some cases (Figure 5), the crack path did not always stay perpendicular to the maximum tensile stress. Other authors [18] have reported similar behavior, discussing how roughness could affect the crack path in impact samples. This could be attributed to the inherent anisotropy induced by the manufacturing process that introduces local stress increases in different directions. Such analysis is beyond the scope of this paper. Finally, future studies should dry the samples after humidity exposure to check if the samples lose the water bands through FTIR so one can distinguish the contributions of physical aging from chemical aging.

## 5. Conclusions

Nylon PA6 with carbon fiber reinforcement demonstrates a significant capacity for water absorption under 50% humidity conditions. A mass absorption test was used to quantify the water adsorption.. Moisture content increases progressively from 1.03% ± 0.28% at 24 h to 5.6% ± 0.48% after 504 h. FTIR in transmission mode confirmed this water absorption. A gradual increase with humidity exposure was observed in C=O (around 1640 cm^−1^) and O–H (about 3300–3500 cm^−1^) bands. This moisture absorption weakens the material’s polymer matrix, leading to a change in tensile strength, elasticity and impact resistance. The progression of moisture absorption in the material over time fits an exponential model $\%=1.0624e^{0.0035h}$ capturing the material’s response to long-term humidity exposure up to 504 h. However, under the testing conditions (50% humidity atmosphere) saturation point was not reached. This study provides designers with life estimates for mechanical properties under the studied exposures.

Exposure to UV light induces distinct changes in mechanical properties. Initially, the material exhibits hardening, as evidenced by a significant increase in Young’s modulus over time, reaching 505 MPa after 504 h. This hardening is accompanied by increased Shore-D hardness, peaking at 66.8 ± 2.52 Shore-D after 504 h. However, this stiffening corresponds to a sharp reduction in Charpy energy absorption, indicating embrittlement possibly due to photo-oxidative degradation.

Humidity induces gradual softening due to moisture absorption, reducing the material’s mechanical properties over time. In contrast, UV light exposure accelerates surface-level oxidative cross-linking, increasing stiffness and hardness but leading to embrittlement and reduced impact resistance. Both exposure mechanisms induced surface changes, producing a change in volumetric measurements. However, the depth of such modification could not be measured. These results mean that for Onyx fixtures, tools or low-volume components stored or used in humid environments, designers should expect softening and impact-energy loss once mass gain exceeds ~2–3 wt% (after ~1 week of continuous exposure at 50% RH), while components under UV will stiffen and harden losing one-third of their impact capacity over ~500 h of continuous exposure. Therefore, service-life predictions and safety factors must be based on these degraded properties rather than the as-printed values.

The SEM analysis reveals significant degradation in the mechanical integrity of Onyx samples due to UV exposure. At 0 h, the material exhibits ductile failure with visible deformation of filaments and plasticity, indicating good energy absorption. After 504 h, the matrix becomes embrittled, limiting filament deformation and resulting in brittle failure with clean, planar fracture surfaces. This behavior resembles high-speed impact failures, as UV exposure mimics high strain-rate effects by reducing the material’s capacity to dissipate energy.

No statistically significant difference with a 5% uncertainty was found between Strength, Elasticity and Hardness.

## Figures and Tables

**Table 1:** Summary of humidity and temperature tests for related studies.

Reference	Humidity	Temp., °C	UV Wavelength, nm
Humeau [7,8]	10 to 80%	25	N/A
Bergeret [8]	100%	135	N/A
Shirinbayan [14]	100%	20 to 70	N/A
Kikuchi [17]	95%	70	N/A
Guo [25]	100%	60	N/A
Moezzi [21]	N/A	N/A	253.7
Goodwin [26]	N/A	75	295 to 400
Bartosz [27]	N/A	N/A	300
Rajendran [23]	30%	43	340

**Table 2:** Summary of statistical relationships among the measured variables. The symbols are + significant relation; − not significant relation.

	Strength	Elasticity	Hardness	Impact
Strength		+	+	−
Elasticity	+		+	+
Hardness	+	+		+
Impact	−	+	+	

## Data Availability

Data available upon request.
